# Sputtered Porous Li-Fe-P-O Film Cathodes Prepared by Radio Frequency Sputtering for Li-ion Microbatteries

**DOI:** 10.1038/s41598-019-47464-2

**Published:** 2019-08-01

**Authors:** V. A. Sugiawati, F. Vacandio, C. Perrin-Pellegrino, A. Galeyeva, A. P. Kurbatov, T. Djenizian

**Affiliations:** 1 Mines Saint-Etienne, Center of Microelectronics in Provence, Department of Flexible Electronics, F – 13541 Gardanne, France; 2 Aix-Marseille Université, CNRS, Electrochemistry of Materials Research Group, MADIREL, UMR 7246, F-13397 Marseille, Cedex 20 France; 3 Aix-Marseille Université, Institut Matériaux Microélectronique Nanoscience de Provence (IM2NP), Faculté des Sciences, Campus de Saint-Jérôme, Avenue Escadrille Normandie Niemen - Case 142, F-13397 Marseille, Cedex France; 4 Al-Farabi Kazakh National University, Center of Physical-Chemical Methods of Research and Analysis, Tole bi str., 96A, Almaty, Kazakhstan

**Keywords:** Materials for energy and catalysis, Energy storage

## Abstract

The increasing demands from micro-power applications call for the development of the electrode materials for Li-ion microbatteries using thin-film technology. Porous Olivine-type LiFePO_4_ (LFP) and NASICON-type Li_3_Fe_2_(PO_4_)_3_ have been successfully fabricated by radio frequency (RF) sputtering and post-annealing treatments of LFP thin films. The microstructures of the LFP films were characterized by X-ray diffraction and scanning electron microscopy. The electrochemical performances of the LFP films were evaluated by cyclic voltammetry and galvanostatic charge-discharge measurements. The deposited and annealed thin film electrodes were tested as cathodes for Li-ion microbatteries. It was found that the electrochemical performance of the deposited films depends strongly on the annealing temperature. The films annealed at 500 °C showed an operating voltage of the porous LFP film about 3.45 V vs. Li/Li^+^ with an areal capacity of 17.9 µAh cm^−2^ µm^−1^ at C/5 rate after 100 cycles. Porous NASICON-type Li_3_Fe_2_(PO_4_)_3_ obtained after annealing at 700 °C delivers the most stable capacity of 22.1 µAh cm^−2^ µm^−1^ over 100 cycles at C/5 rate, with an operating voltage of 2.8 V vs. Li/Li^+^. The post-annealing treatment of sputtered LFP at 700 °C showed a drastic increase in the electrochemical reactivity of the thin film cathodes vs. Li^+^, leading to areal capacity ~9 times higher than as-deposited film (~27 vs. ~3 µAh cm^−2^ µm^−1^) at C/10 rate.

## Introduction

Li-ion microbatteries have emerged a new direction for powering the miniaturized modern devices, for instance RFID tags, implantable medical devices, microsensors, and smart cards. In order to advance towards the fabrication of all-solid-state thin film microbatteries, numerous studies have been reported to find innovative cathode and anode materials. Most of reports have been largely devoted to anode materials such as self-organized TiO_2_ nanotubes^1,2^, silicon nanowires^3^, Cu_2_Sb^4^, aluminum nanorods^5^, carbon nanotubes^6,7^, and LiNiVO_4_^8^. Besides the anode materials, exploiting the cathode materials are very crucial to realize the fabrication of full cell Li-ion microbatteries. Iron-based phosphate especially olivine-type LiFePO_4_ (LFP) becomes a popular cathode as it is known to be cheap, abundant, low toxic, and thermally stable^9^. The strong Fe-O covalent bonds in LFP cathodes has such a special characteristic compared to layered LiCoO_2_-type materials^10^. Indeed, this covalent bond greatly improve the stability of O in the lattice, thus increasing the reliability of the materials.

In the field of thin-film microbatteries, study the properties of the pure active layers deposited onto the current collectors is essential since neither binder nor conductive additive are utilized^11–13^. By using the thin film cathodes, the intrinsic drawbacks such as a low electronic conductivity and low Li^+^ diffusion mobility can also be suppressed because the thickness of the cathode material is considerably reduced^14–16^. Much studies have reported the growth of thin-film electrodes by various techniques such as pulsed laser deposition (PLD)^17–22^, sputtering^23,24^ and sol-gel^25–27^. Among these different approaches, radio frequency (RF) sputtering technique is considered to be the most versatile way to fabricate thin-film cathodes^16,28^ because the sputtering parameters are easily controlled to obtain a good quality film, such as sputtering power, argon pressure, and substrate temperature during deposition.

More recently, RF sputtering have attracted substantial attention in the fabrication of the flexible and transparent thin-film batteries. For example, Lee *et al*.^29^ reported a flexible battery applied on contact lens using LFP cathode material deposited on the flexible polyimide substrate by RF sputtering, the thin film reached 63.8% of the theoretical capacity without carbon coating and the cell showed stable performance under wet conditions. Also, Oukassi *et al*.^30^ deposited transparent thin film batteries layers using RF sputtering, a discharge capacity of 0.15 mAh at C/2 rate with an average capacity loss of solely 0.15% per cycle can be obtained.

Due to a strong research interest focused on searching for lithium transition metal phosphates as cathode materials, NASICON type-Li_3_Fe_2_(PO_4_)_3_ has been reported of having a high lithium mobility and can intercalate up to two moles of lithium within the structure^31^. Besides a theoretical capacity of 128 mAh g^−1^ ^32^, Li_3_Fe_2_(PO_4_)_3_ cathode is currently attracting considerable interest due to several advantages such as low toxicity, low cost, good ionic conductivity, and large natural abundance^33^. Furthermore, the structure of the NASICON type-Li_3_Fe_2_(PO_4_)_3_ is more stable than that of olivine-type LiFePO_4_^34^ and its synthesis is easier as it can be prepared directly in air. NASICON type-Li_3_Fe_2_(PO_4_)_3_ have been commonly synthesized via various methods such as solid-state reactions^35^, hydrothermal^36^, sol-gel combustion^32^ and ultrasonic spray pyrolysis^37^. However, there are only limited reports regarding the Li_3_Fe_2_(PO_4_)_3_ film deposited by RF Sputtering^12^. In the present work, we propose to study the effect of annealing temperature on RF sputtered LFP thin-films in terms of morphology, composition, structure, and electrochemical properties. We show that the thermal treatment performed at 700 °C in air leads to the formation of porous Li_3_Fe_2_(PO_4_)_3_ showing the best electrochemical performance and therefore being a good candidate as a cathode for Li-ion microbatteries.

## Results and Discussion

### Structural and chemical characterization

**Figure 1:**
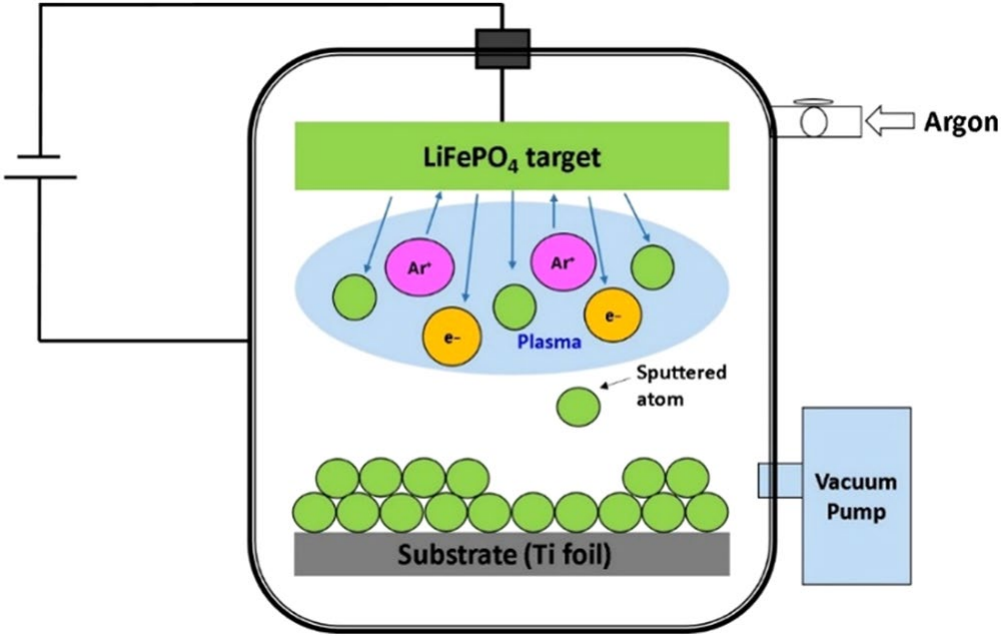
Schematic representation of a RF sputtering system.

The schematic representation of the sputtering system is illustrated in Fig. 1. The sputtering process can be briefly described as follows: an inert argon gas is introduced in the vacuum chamber and the ionization process leads to the formation of a plasma. Then, the energized ions hitting the LFP target (cathode) eject out adatoms that are deposited on the substrate to form a thin film. Various parameters such as the nature of the substrate, the sputtering power, the argon pressure, the duration, and the deposition temperature have significant influences on the growth rate and features of the deposited film^14^.

**Figure 2:**
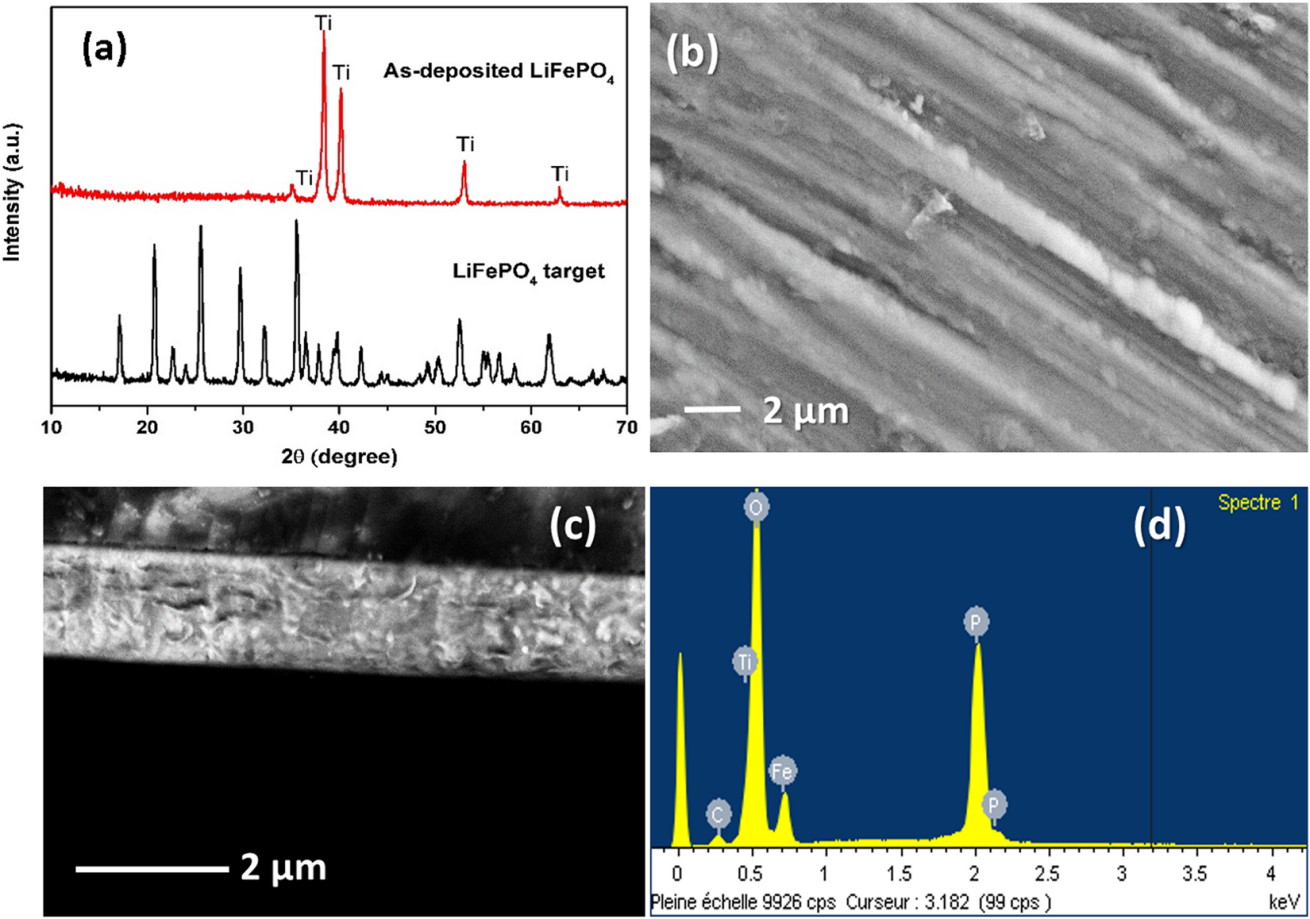
XRD patterns of the commercial LFP target and as-deposited LFP film (**a**), SEM images of as-deposited LFP film from the top view (**b**), and cross-sectional view (**c**) and EDX analysis of the as-deposited LFP film (**d**).

In this work, a sputtering power of 3 W cm^−2^ and argon pressure of 7 mTorr during 12 hours of deposition was employed to obtain smooth and dense layers as well as a better adhesion between the films and the substrates. Owing to a low deposition rate, 12 hours of deposition is required to obtain LFP films with a sufficient thickness for the targeted application. The XRD pattern of the commercial LFP target and as-deposited film are plotted in Fig. 2a. Obviously, the initial target has well-defined peaks corresponding to the Olivine LiFePO_4_ (JCPDS File No. 040-1499). However, after deposition at room temperature, only Ti peaks can be identified suggesting that the material is amorphous. The amorphous nature of the thin films deposited without heating treatment, either substrate heating during deposition or post-annealing have been reported in previous studies^12,14^. Thus, the thermal treatment step is essential because the crystalline materials are known for their better electrochemical reactivities versus Li ions^38^.

**Figure 3:**
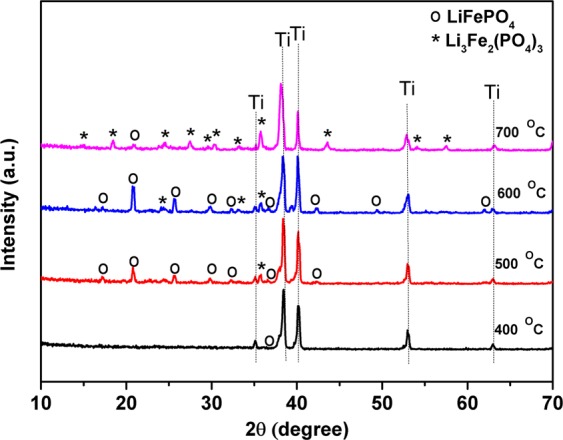
XRD patterns of LFP thin films annealed at various temperatures in air for one hour.

Figure 2b,c display the SEM images of the thin films deposited at 3 W cm^−2^ W during 12 hours. As-deposited film shows a dense layer with a thickness of approximately 1.6 µm that corresponds to a growth rate of 2.2 nm min^−1^. EDX analysis after deposition (Fig. 2d) revealed a Fe:P atomic ratio of 14.33(1): 14.58(1), which is in agreement with the LFP formula. To further study in detail the effects of the annealing temperature, the as-deposited LFP films were annealed at 400 °C, 500 °C, 600 °C, and 700 °C, respectively in air atmosphere for one hour. In agreement with the previous studies, as-deposited films show poor crystallinity. As the films are annealed, the adsorbed atom on the surface of the films are activated and rearranged to form ordered crystal structure^39^. The XRD patterns for the LFP films are gathered in Fig. 3 for analyzing the effects of the thermal treatment at various temperatures on their crystal structure. At first glance, the typical peaks ascribable to crystalline LFP and Li_3_Fe_2_(PO_4_)_3_ phases can be detected at elevated temperatures. The amorphous nature of LFP is evidenced for the film annealed at 400 °C since only Ti peaks are visible. This result confirms that annealing temperature higher than 400 °C is required to crystallize LFP thin film^13^. As the temperature was increased to 500 °C, several characteristic peaks of 2*θ* suited at 17.21°, 20.82°, 25.65°, 29.87° and 32.36° marked as symbol (o) are attributed to the (0 2 0), (0 1 1), (0 2 1), (1 2 1) and (0 3 1) planes of LFP phase. Besides the presence of LFP peaks, two small Li_3_Fe_2_(PO_4_)_3_ peaks with low intensities marked as symbol (*) are also detected at 2*θ* of 24.4° and 35.8° suggesting that the decomposition of LiFePO_4_ phase has also started. Nevertheless, crystalline LFP seems to be the most predominant phase at 500 °C. This is probably due to a slow heating rate that is applied during annealing treatment. As reported elsewhere^40^, the heating rate plays an important role in decelerating a second phase formation. It is important to note that in our work the films were heated at 2 °C min^−1^ to avoid the fast formation of Li_3_Fe_2_(PO_4_)_3_ phase due to the LFP sensibility to air atmosphere. Thus, the LFP phase can be clearly detected at 500 °C after annealing in air atmosphere. Presumably, Li_3_Fe_2_(PO_4_)_3_ phase might be quickly obtained when the heating rate is faster than 2 °C min^−1^ in air. When the annealing temperature further raised to 600 °C, more Li_3_Fe_2_(PO_4_)_3_ (JCPDS file no. 047-0107) peaks have appeared. The annealed films at 600 °C are assumed to be composed of mixed LFP and Li_3_Fe_2_(PO_4_)_3_ phases. In a good agreement with the previous reports^17,18,24,40^, the optimum annealing temperature for the crystallization of LFP was 500 °C. As expected, by increasing the annealing temperature up to 700 °C, almost all phases are transformed to Li_3_Fe_2_(PO_4_)_3_ due to the oxidation of Fe^2+^ by oxygen from air according to Eq. (1) ^12^.

1 $$12{{\rm{LiFePO}}}_{4}+3{{\rm{O}}}_{2}\to 4{{\rm{Li}}}_{3}{{\rm{Fe}}}_{2}{({{\rm{PO}}}_{4})}_{3}+2{{\rm{Fe}}}_{2}{{\rm{O}}}_{3}$$

Another LFP phase with very low intensity at 2*θ* = 20.82° is detected as minor phase. However, the peak intensity appears to be weakening at this temperature. As previously reported^41^, encouraging results on the electrochemically active Li_3_Fe_2_(PO_4_)_3_ phase that obtained after annealing at 700 °C have been studied in the form of composite electrodes carrying polymer binder and carbon additive. NASICON-type Li_3_Fe_2_(PO_4_)_3_ can be considered as an interesting cathode material for Li-ion batteries by hosting about 2 additional Li^+^ (Fe^3+^/Fe^2+^ redox couple) with great reversibility at a voltage plateau of around 2.8 V^23–27^. However most reports on this material focus on composite Li_3_Fe_2_(PO_4_)_3_ cathodes prepared by slurry coatings instead of thin films.

**Figure 4:**
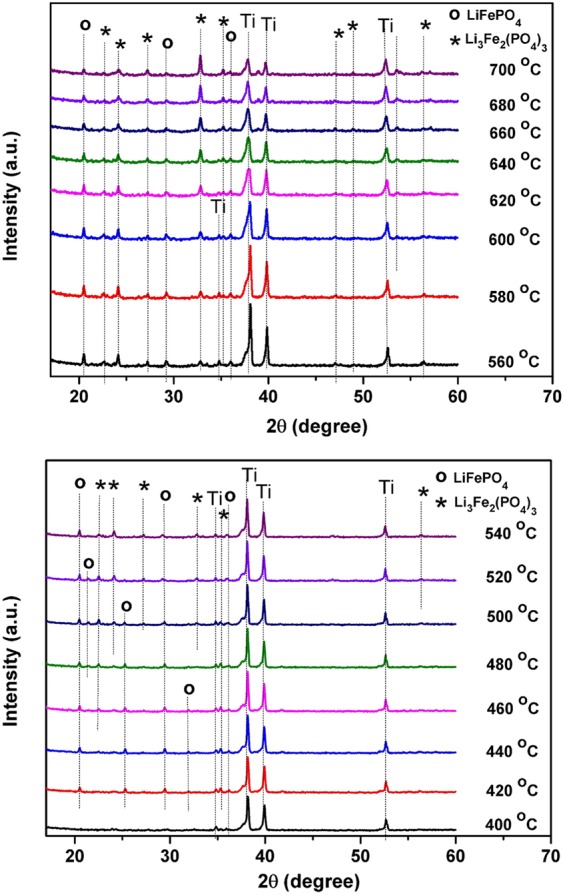
*In-situ* XRD patterns (λ = 1.54 Å) of as-deposited LFP film during thermal annealing performed between 400 and 700 °C by step of 20 °C.

In-depth structural studies have been also carried out by *in-situ* XRD experiments. The purpose of this particular structural analysis is to follow the evolution of the formed phases at elevated temperature after deposition time of 3 hours. The different XRD patterns given in Fig. 4 were acquired during annealing process between 400 °C and 700 °C using steps of 20 °C. Starting from 400 °C, a low intensity peak corresponding to LFP appeared at 2*θ* = 35.90°. In fact, Li_3_Fe_2_(PO_4_)_3_ starts to crystallize at 420 °C which is shown by the presence of a small peak at 2*θ* = 35.36° with a low crystallinity. Both Li_3_Fe_2_(PO_4_)_3_ and LFP peaks can be clearly observed in the temperature range between 560 °C and 640 °C, mainly the stronger peak intensity located at 2*θ* = 20.82° for LFP phase and 2*θ* = 24.14° for Li_3_Fe_2_(PO_4_)_3_ phase. Then, the intensities of both peaks are getting weaker and even disappeared when annealing temperatures are higher than 640 °C or lower than 560 °C. This temperature range is considered to be less preferred for the Li ion intercalation/de-intercalation in the cathode materials. At the temperature below 560 °C, the presence of LFP phase is more pronounced, on the contrary when the annealing temperature is higher than 640 °C the appearance of Li_3_Fe_2_(PO_4_)_3_ phase becomes predominant. Similar results on the LFP powders were observed by Hamelet *et al*.^41^ and Delacourt *et al*.^42^, demonstrating the significant decomposition of the LFP phase occurred on thermal treatment at temperatures greater than ~ 500 °C and the progressive sharpening peaks for the Li_3_Fe_2_(PO_4_)_3_ can be observed afterwards. Bünting *et al*.^43^ reported that the Ti interdiffusion occured during the crystallization process of the LFP thin film, leading to the LiTi_2_(PO_4_)_3_ (LTP) phase formation. From the XRD pattern, the reflection peaks at 2*θ* = 20.82° and 2*θ* = 24.4° which overlap with the reflection peaks of LFP and Li_3_Fe_2_(PO_4_)_3_ are characteristics for LTP phase^44^.

**Figure 5:**
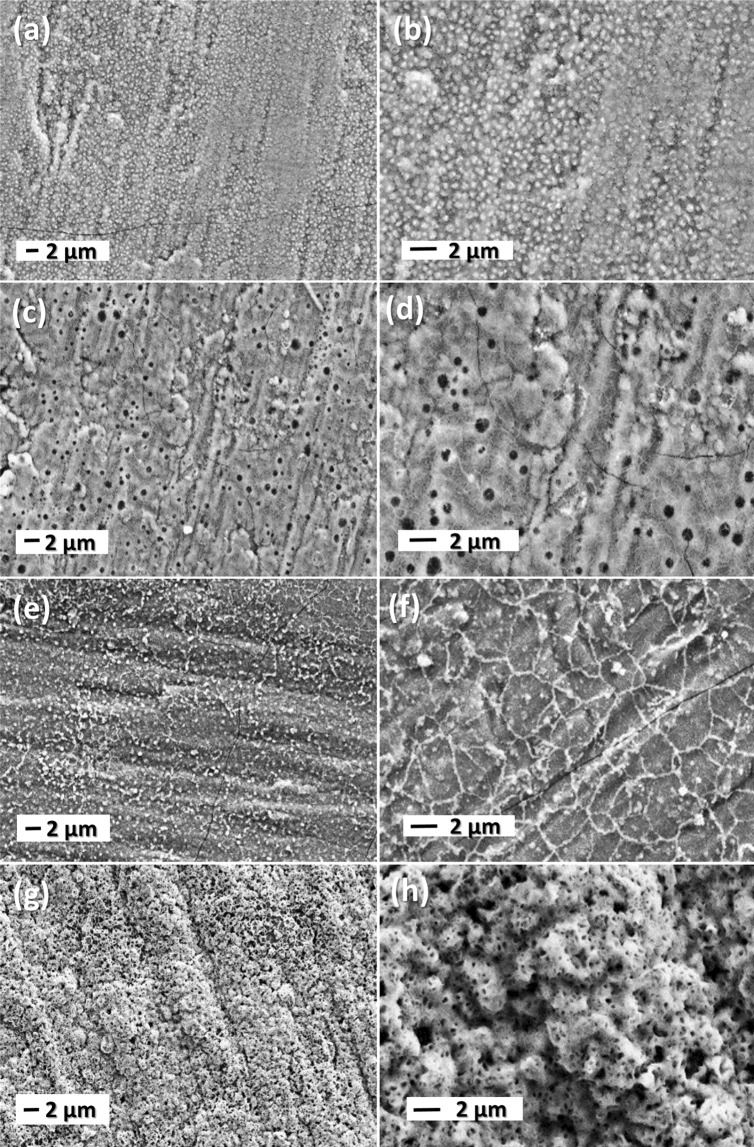
SEM images the annealed films at different temperatures LFP-400 (**a**,**b**), LFP-500 (**c**,**d**), LFP-600 (**e**,**f**) and LFP-700 (**g**,**h**).

As the electrochemical performance of electrodes are often driven by their morphology, the examination of thin-films was performed by SEM. Figure 5 shows the surface of the annealed thin films. Apparently, the roughness increases with increasing of the annealing temperature.

According to the SEM examinations, LFP-400 is composed of small grains and exhibits a rough surface due to an inhomogeneous size distribution of particles (Fig. 5a,b). As the temperature is raised to 500 °C, the surface is characterized by the presence of large pores with various diameters (Fig. 5c,d), suggesting the formation of crystalline LFP phase^40,45^. As the temperature reaches 600 °C, the microstructured deposit is highlighted by appearance of grain boundaries with various grain sizes. After annealing at 700 °C, the surface becomes coarse and highly porous, which is consistent with the formation of the NASICON-type Li_3_Fe_2_(PO_4_)_3_ phase^12^. From the above results, it is interesting to note that the texture of LFP thin films shows some cracks except for the sample annealed at the highest temperature.

### Electrochemical characterization

**Figure 6:**
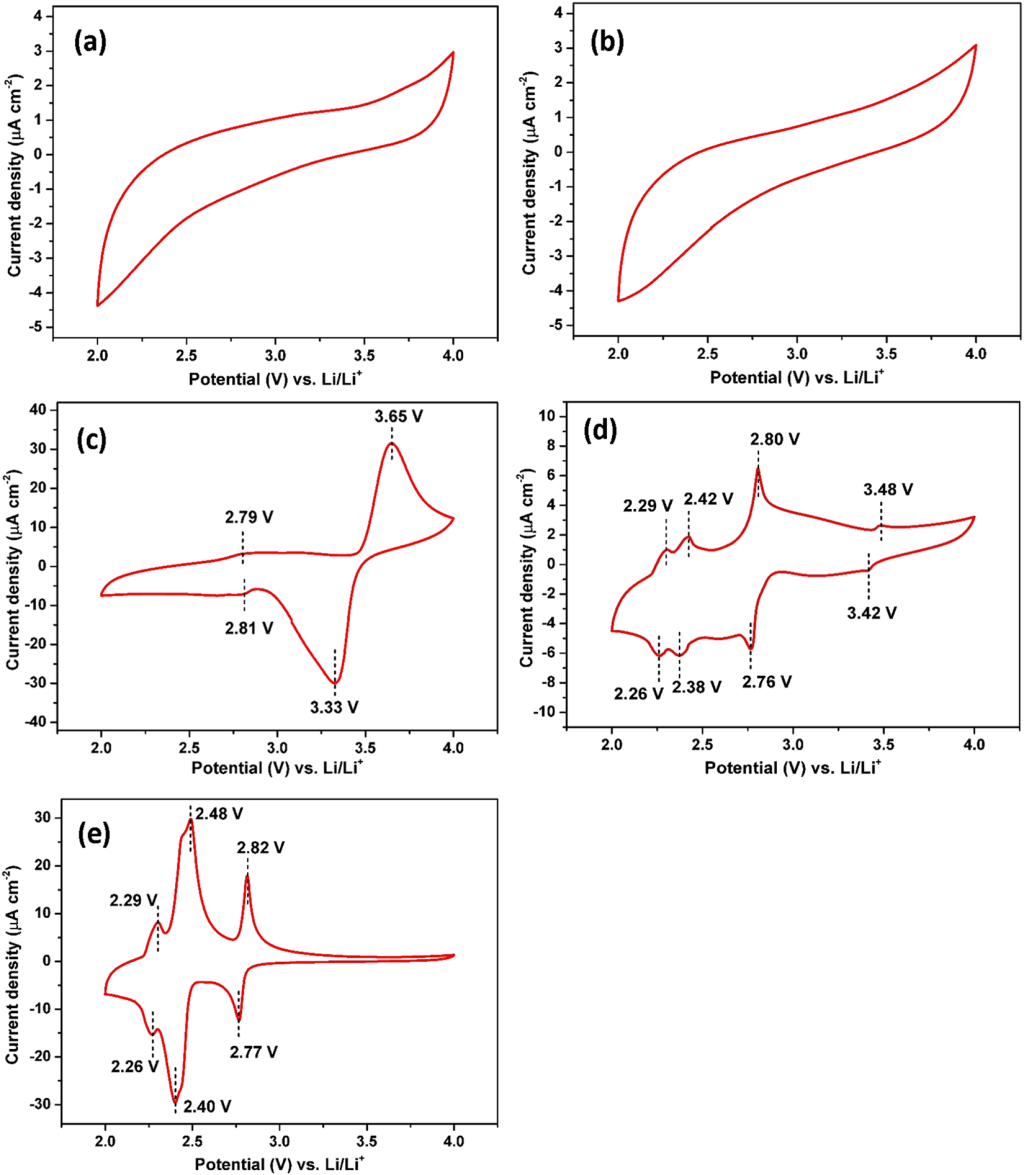
Cyclic voltammograms (1^st^ cycle) of the LFP-0 film (**a**), LFP-400 (**b**), LFP-500 (**c**), LFP-600 (**d**) and LFP-700 (**e**).

Cyclic voltammetry (CV) experiments were performed to evaluate the influence of the annealing process on the reactivity of LFP films with Li^+^. Figure 6 shows the voltammograms obtained from the first cycle. No redox peak can be identified for LFP-0 (Fig. 6a) confirming that amorphous LFP is not electrochemically active. A similar behavior is observed for LFP-400 (Fig. 6b) suggesting that amorphous LFP is predominant despite the detection of a small peak by *in-situ* XRD which is attributed to the crystalline LFP phase. The LFP-500 film showed a well-defined peak pair and reversible redox peaks at 3.65 V and 3.33 V vs. Li/Li^+^ corresponding to the anodic and cathodic peaks, respectively (Fig. 6c). These peaks are typical for the oxidation and reduction reactions of olivine-type LiFePO_4_ material with Li^+^ according to Eqs (2) and (3)^46^.

2 $${\rm{Oxidation}}:\,{{\rm{LiFePO}}}_{4}-{{\rm{xLi}}}^{+}-{{\rm{xe}}}^{-}\to {{\rm{xFePO}}}_{4}+(1-{\rm{x}}){{\rm{LiFePO}}}_{4}$$

3 $${\rm{Reduction}}:\,{{\rm{FePO}}}_{4}+{{\rm{xLi}}}^{+}+{{\rm{xe}}}^{-}\to {{\rm{xLiFePO}}}_{4}+(1-{\rm{x}}){{\rm{LiFePO}}}_{4}$$

The low potential difference between the oxidation and reduction peaks (∆Ep) about 0.32 V vs. Li/Li^+^ indicates that the film reveals a good electrochemical reversibility for Li^+^ intercalation/de-intercalation. In addition, a broad peak pair with very low absolute current densities can also be observed at 2.79 V for the oxidation peak and 2.81 V for the reduction peak. These peaks correspond to the reaction of Li ions with the Li_3_Fe_2_(PO_4_)_3_ phase.

As the annealing temperature increases from 500 °C to 600 °C, a small oxidation peak at 3.48 V and a reduction peak at 3.42 V which are attributed to the two-phase transformation of the (Fe^3+^/Fe^2+^) redox couple due to the reversible intercalation of Li ions in the LFP crystal structure (Fig. 6d) are progressively vanished. This result is in good agreement with the XRD data showing that the LFP phase is clearly observable at 600 °C while the complete transformation into Li_3_Fe_2_(PO_4_)_3_ phase has yet to be occurred. In addition, a pair of anodic and cathodic peaks at around 2.29 V and 2.26 V vs. Li/Li^+^ could be attributed to the formation of the iron tavorite phase LiFePO_4_(OH)^12,47–49^ according to Eq. (4)

4 $$2{{\rm{LiFePO}}}_{4}+2{{\rm{H}}}_{2}{\rm{O}}\to 2{{\rm{LiFePO}}}_{4}({\rm{OH}})+{{\rm{H}}}_{2}$$

Thin film annealed at 700 °C (Fig. 6e) reveals a peak pair, a reduction peak at 2.77 V and an oxidation peak at 2.82 V which are attributed to the reversible intercalation reactions of Li^+^ ions into Li_3_Fe_2_(PO_4_)_3_ as described by Eq. (5)^12^.

5 $${{\rm{Li}}}_{4}{{\rm{Fe}}}_{2}{({{\rm{PO}}}_{4})}_{3}={{\rm{Li}}}^{+}+{{\rm{Li}}}_{3}{{\rm{Fe}}}_{2}{({{\rm{PO}}}_{4})}_{3}+{\rm{e}}-$$

The cathodic and anodic peaks are symmetric and have similar current density values, indicating the excellent degree of reversibility. In a good agreement with the XRD analysis, due to the interdiffusion between the Ti and the deposited LFP thin film, the additional cathodic peak at 2.38 V and anodic peak at 2.42 V are observable at 600 °C and 700 °C which are characteristics for the LiTi_2_(PO_4_)_3_^44^ phase, according to Eq. (6)

6 $${{\rm{Li}}}_{3}{{\rm{Ti}}}_{2}{({{\rm{PO}}}_{4})}_{3}={{\rm{LiTi}}}_{2}{({{\rm{PO}}}_{4})}_{3}+2{{\rm{Li}}}^{+}+2{{\rm{e}}}^{-}$$

**Figure 7:**
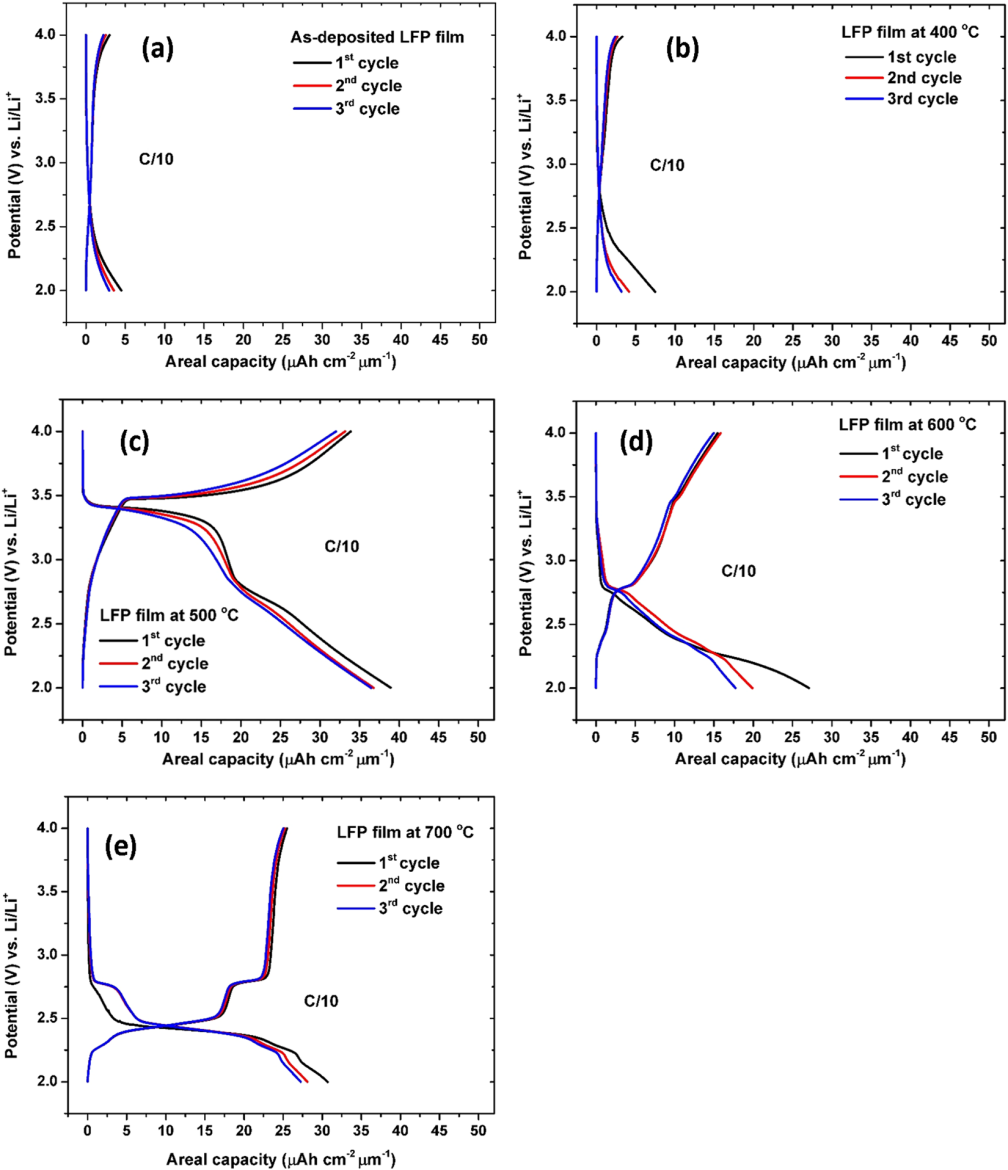
Charge-discharge profiles of the LFP-0 film (**a**), LFP-400 film (**b**), LFP-500 film (**c**), LFP-600 film (**d**) and LFP-700 film (**e**).

Preliminary CV results highlight that LFP thin films performances are strongly related to their structure and morphology. Indeed, the capacities of thin films prepared under different conditions varied significantly. Figure 7 shows the typical charge-discharge profiles of the five cathode thin films in the potential range 2–4 V at a current rate of C/10. In accordance with the CV curves, no voltage plateau can be observed for LFP-0 and LFP-400, delivering very low capacity values (<10 µAh cm^−2^ µm^−1^). More remarkably, a couple of defined charge-discharge plateaus at 3.3 and 3.5 V are clearly seen at 500 °C corresponding to the reversible intercalation of lithium ions in crystallized LiFePO_4_. These charge-discharge plateaus are fully consistent with the peaks of the CV curves. In many previous reports, crystalline LFP films can be obtained after annealing under inert atmosphere. In this work, the presence of the LFP phases can be detected after annealing treatment in air atmosphere. This result suggests that the phase transformation is slower, probably due to the low heating rate applied during the annealing treatment as previously mentioned. The capacities values for the 1^st^, 2^nd^, and 3^rd^ cycles are 39 µAh cm^−2^ µm^−1^, 37 µAh cm^−2^ µm^−1^ and 37 µAh cm^−2^ µm^−1^ with the corresponding coulombic efficiencies of 88%, 90% and 88%, respectively. The slight decrease of the coulombic efficiency is probably due to the small amount of Li_3_Fe_2_(PO_4_)_3_ phase that has been evidenced by XRD analysis.

For LFP-600, the voltage plateau around 2.8 V is ascribed to the reaction implying the Li_3_Fe_2_(PO_4_)_3_ phase. The presence of a short plateau at 3.5 V can be attributed to the low amount of crystalline LFP. The capacities of the LFP film annealed at 600 °C for the 1^st^, 2^nd^, and 3^rd^ cycles are 27 µAh cm^−2^ µm^−1^, 20 µAh cm^−2^ µm^−1^ and 18 µAh cm^−2^ µm^−1^ and the coulombic efficiencies are 57%, 80% and 84%, respectively. The low coulombic efficiency obtained in the 1^st^ cycle could be explained by the mixed phases. In addition, the LFP film annealed at 700 °C revealed a well-defined plateau around 2.4 V which indicates the Li ions insertion into the LiTi_2_(PO_4_)_3_ lattice structure and another plateau appeared at 2.8 Vcorresponds to redox reactions involving the Li_3_Fe_2_(PO_4_)_3_ phase^50–52^. The capacities of the film for the 1^st^, 2^nd^, and 3^rd^ cycles are 31 µAh cm^−2^ µm^−1^, 28 µAh cm^−2^ µm^−1^ and 27 µAh cm^−2^ µm^−1^, while the associated coulombic efficiencies can reach 83%, 90% and 92%, respectively. Beyond the influence of the chemical and structural composition, the enhanced electrochemical properties of LFP-500 and LFP-700 can be explained by the porosity of the both layers. Actually, it has been reported that the use of porous electrodes can improve the electrochemical performance of batteries owing to the larger electrode/electrolyte interface^53,54^. In the present case, the porous nature of the LFP-500 and LFP-700 films is supposed to promote the penetration of the electrolyte providing more reaction electrochemical actives sites for the lithium ions intercalation/de-intercalation owing to larger accessible surface of the thin films. This is beneficial to improve the Li ion exchange between the LFP film electrode and electrolyte because the pores serve as channels for fast lithium supply^55^.

**Figure 8:**
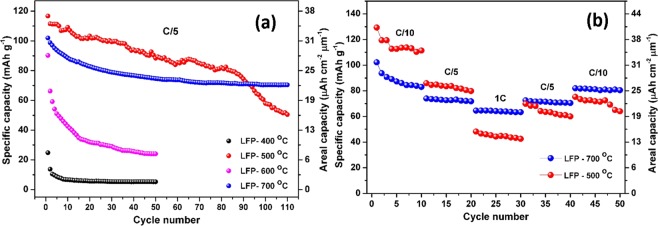
Long-term cycling stability study showing capacity versus the cycle number at C/5 rate for the various annealed LFP thin films (**a**), and rate capability of LFP-500 and LFP-700 films at multiple C-rates (**b**).

In order to have a better insight into the potential use of annealed LFP layer as cathode materials, rate capability and long-term cycling tests were carried out. Figure 8a confirms that LFP-400 film provides the lowest capacity values attaining only 2 µAh cm^−2^ µm^−1^ at the 50^th^ cycle. LFP-500 film exhibits the highest discharge capacities up to the 90^th^ cycle but this cathode is subjected to a continuous capacity loss with a drop after the 80^th^ cycle. The capacities obtained at the 1^st^, 2^nd^, and 100^th^ cycle are 36 µAh cm^−2^ µm^−1^, 35 µAh cm^−2^ µm^−1^, and 18 µAh cm^−2^ µm^−1^, respectively. Thus, only half of the initial capacity is retained after 100 cycles. It can be noticed that similar behavior regarding the capacity fading has been reported for the annealed LiNi_1/3_Co_1/3_Mn_1/3_O_2_ thin films at 500 °C^22^. Such a low cycling stability may be ascribed to the poor adhesion strength between the thin film and the substrate that are combined to the presence of cracks, leading to the detachment of the layer from the current collector and the subsequent loss of electrical contact. For the LFP-600 film, the first discharge capacity is relatively high (29 µAh cm^−2^ µm^−1^) but severely drops to reach only 8 µAh cm^−2^ µm^−1^ after the 50^th^ cycle. This behavior could be attributed to the presence of cracks and mixed unstable LiFePO_4_ and Li_3_Fe_2_(PO_4_)_3_ phases. Finally, LFP-700 film shows the best cycling stability. The capacities obtained for the LFP-700 were found to be 32 µAh cm^−2^ µm^−1^ for the first cycle and 22 µAh cm^−2^ µm^−1^ for the 100^th^ cycle corresponding to a capacity retention of around 70%. The good storage performance and the stability of the LFP-700 film could be mainly attributed to feature properties of the Li_3_Fe_2_(PO_4_)_3_ thin film. Compared to LFP-700 sample, the sharp capacity dropping of LFP-400, LFP-500 and LFP-600 in the first few cycles can be imputed to the cracks that are responsible for the poor mechanical properties. For more details, the areal capacity values and the capacity retention at the 50^th^ and 100^th^ cycles are summarized in Table 1.

**Table 1:** Electrochemical properties of LFP films annealed at various temperatures.

	400 °C	500 °C	600 °C	700 °C
Discharge capacity of the 1^st^ cycle (µAh cm^−2^ µm^−1^)	8	36	29	32
Discharge capacity of the 50^th^ cycle (µAh cm^−2^ µm^−1^)	2	28	8	24
Discharge capacity of the 100^th^ cycle (µAh cm^−2^ µm^−1^)	—	18	—	22
Capacity retention (%) 1^st^–50^th^ cycle	25	78	29	75
Capacity retention (%) 1^st^–100^th^ cycle	—	50	—	70

As a further examination, we also studied the rate capability of the thin films annealed at 500 °C and 700 °C because they showed the most interesting electrochemical properties (Fig. 8b). LFP-500 delivers stable capacity values of 35 µAh cm^−2^ µm^−1^ at C/10 rate, 25 µAh cm^−2^ µm^−1^ at C/5 rate, and 13 µAh cm^−2^ µm^−1^ at 1C rate. Although the initial C/10 and C/5 discharge capacity are superior compared to LFP-700 film, the LFP-500 shows relatively larger degradation in capacity with further increasing discharge current. Indeed, significant capacity losses of 28% and 48% are observed for subsequent tests at C/5 and 1C rate, respectively. The LFP-700 film cathode delivers capacity values of 25 µAh cm^−2^ µm^−1^ at C/10 rate, 22 µAh cm^−2^ µm^−1^ at C/5 rate, and 19 µAh cm^−2^ µm^−1^ at 1C rate. The capacity slightly decreased of only 11% from C/10 to C/5 rate and decreased of about 15.5% from C/5 to 1C rate. More remarkably, the discharge capacity values of about 22 µAh cm^−2^ µm^−1^ and 25 µAh cm^−2^ µm^−1^ at C/5 and C/10 show that the full capacity is completely recovered even after fast cycling tests. Therefore, it is found that the highest annealing temperature treatment (700 °C) is able to strongly improve the electrochemical stability of LFP films electrodes, especially in the rapid charge-discharge regimes. After few initial cycles, LFP-700 film reveals high reversibility and prominent cycling stability. The better performance of LFP-700 film is also attributed to the presence of the LTP phase as a good ionic conductor^56^.

To sum up, the amorphous and partially crystalline LFP phases may not be stable during Li-ion intercalation/de-intercalation, yielding in a fast capacity fading^22^. Thus, through this study, we highlight the important role of the post annealing treatment mainly for the intercalation cathodes. This treatment is responsible for the film texturation, thus it could help minimizing ionic limitation by enhancing the contact area between electrolyte and the film surface contact, as well as could enhance the electrochemical activity^54,57–59^.

## Conclusion

In summary, we have successfully prepared porous thin film cathodes by RF sputtering technique and subsequent annealing treatment in air atmosphere. The electrochemical performance of the thin films depends strongly on the annealing conditions. Different morphological and structural properties were obtained by varying the temperature treatment, leading to a significant influence on the cell performances. We report that annealing at 500 °C is required to obtain porous olivine type-LiFePO_4_ film even if the electrochemical tests revealed a drop of capacities after the 80^th^ cycle. Complete transformation from porous olivine type-LiFePO_4_ to porous NASICON-type Li_3_Fe_2_(PO_4_)_3_ occurring at 700 °C led to a remarkable stability of the electrode with good electrochemical performance even at fast kinetics.

## Methods

### Preparation of LFP thin films

Thin films were deposited on Ti foil substrate by radio frequency sputtering in a Plassys MP 300 apparatus at room temperature. In the present work, radio-frequency (RF) sputtering is utilized to deposit LFP layers from a commercial target. The target was LiFePO_4_ (LFP) purchased from Neyco, purity 99.9%. Prior to their insertion in the deposition chamber, Ti foils were cleaned by ultrasonication in acetone, 2-propanol, and methanol for 10 minutes each. Pre-sputtering of the targets before deposition was employed to remove the native oxide layer. Before deposition, vacuum was applied into the sputtering chamber until the pressure was around 3 × 10^−6^ mbarr. Sputtering was performed in a pure argon atmosphere. The argon pressure was set to 7 mTorr and the applied RF sputtering power was fixed to 75 W (3 W cm^−2^). Deposition time was adjusted for 12 hours. The post-annealing treatment was carried out at various temperatures (400 °C, 500 °C, 600 °C, and 700 °C) for one hour with a heating rate of 2 °C min^−1^ (Furnace: NABERTHERM Controller B 180). The as-deposited thin film was labelled as LFP-0 and post-annealed thin films at 400 °C, 500 °C, 600 °C and 700 °C were labelled as LFP-400, LFP-500, LFP-600, and LFP-700, respectively.

### Materials characterization

The crystalline structure of the initial LiFePO_4_ target and as-deposited film were analyzed by X-ray diffraction in a D5000 BRUKER-SIEMENS diffractometer with Cu-Kα radiation (wavelength = 1. 5406 Å) over 2θ range of 10–70°. The accelerating voltage and current were 40 kV and 30 mA, respectively and a scan speed of 0.04° per second was utilized. The *in-situ* X-ray diffraction measurement was also performed on an X’Pert diffractometer with Cu-Kα radiation. As-deposited LFP film was mounted on an adapted furnace and annealed from 400 °C to 700 °C with an interval of 20 °C. The data were collected every 300 s with scan-step size 0.04°. The diffractogramms were analyzed using JCPDS-ICDD (Joint Committee on Powder Diffraction Standards-International Center for Diffraction Data). Morphological properties of the thin films were characterized by Scanning Electron Microscopy (SEM) using a field-emission scanning electron microscope (Ultra-55 Carl Zeiss).

### Electrochemical measurements

All cells were assembled in an argon-filled glove box (MBraun, Germany) with H_2_O and O_2_ contents were less than 0.5 ppm. The electrochemical properties of the LFP thin films (LFP-0, LFP-400, LFP-500, LFP-600, and LFP-700) were evaluated by cyclic voltammetry (CV) and chronopotentiometry using a VMP3 potentiostat (Bio Logic, France). For the electrochemical tests, two-electrode Swagelok cells were assembled using the LFP thin films as the working electrode while metallic lithium foils served as both the counter and the reference electrodes. It is important to specify that the electrodes were not mixed with any additive and binder. The working and counter electrodes were separated by a Whatman glass microfiber soaked in the liquid organic electrolyte composed of 1 mol L^−1^ of LiPF_6_ in ethylene carbonate (EC) – diethyl carbonate (DEC) (1:1 in w/w). The area of the LFP films was 0.49 cm^2^ and the average thickness of the film was ca. 1.6 µm, measured from cross section SEM images. The CV measurement was performed in the potential range between 2 and 4 V at a scan rate of 0.1 mV s^−1^ and galvanostatic cycling with potential limitation (GCPL) was performed in the potential range in the same potential window at multiple C-rates (C/10, C/5, C/2 and 1C). The charge or discharge rate denoted as C/*n* means that the battery is fully charged or discharged up to its total storage capacity in *n* hours, whilst C-rate of the LFP films were calculated by estimating the weight of active material in the thin film from the film thickness and the density of 3.6 g cm^−3^.
